# Topological Frontier-Based Exploration and Map-Building Using Semantic Information

**DOI:** 10.3390/s19204595

**Published:** 2019-10-22

**Authors:** Clara Gomez, Alejandra C. Hernandez, Ramon Barber

**Affiliations:** Roboticslab Research Group, Department of Systems and Automation, University Carlos III of Madrid, 28911 Madrid, Spain; alejhern@ing.uc3m.es (A.C.H.); rbarber@ing.uc3m.es (R.B.)

**Keywords:** robot exploration, frontier-based exploration, indoor environments, loop closure, mobile robot navigation, topological map

## Abstract

Exploration of unknown environments is a fundamental problem in autonomous robotics that deals with the complexity of autonomously traversing an unknown area while acquiring the most important information of the environment. In this work, a mobile robot exploration algorithm for indoor environments is proposed. It combines frontier-based concepts with behavior-based strategies in order to build a topological representation of the environment. Frontier-based approaches assume that, to gain the most information of an environment, the robot has to move to the regions on the boundary between open space and unexplored space. The novelty of this work is in the semantic frontier classification and frontier selection according to a cost–utility function. In addition, a probabilistic loop closure algorithm is proposed to solve cyclic situations. The system outputs a topological map of the free areas of the environment for further navigation. Finally, simulated and real-world experiments have been carried out, their results and the comparison to other state-of-the-art algorithms show the feasibility of the exploration algorithm proposed and the improvement that it offers with regards to execution time and travelled distance.

## 1. Introduction

Autonomous robot navigation needs the existence of a map or representation where the robot can identify its position and the elements concerned with the application to perform. In the luckiest case, the robot will be provided with an a priori map with all the required information. However, in most cases, the robot will not have any prior information of the environment and it will have to build the representation by itself through exploration strategies. Exploration is a fundamental problem to guarantee the autonomy of a robot. It deals with autonomously discovering an unknown area while acquiring the most important information for the desired application. Commonly, exploration and map-building strategies are related as the robot can build a representation of the environment while it explores (although not all of the exploration applications require mapping). An exploration strategy finishes when all the environment is explored or the goal of the application is reached.

Exploration mainly deals with the question: Given the current knowledge of the environment, where should I move next to acquire the maximum information of the unknown environment? One of the most common strategies to answer to this question is Frontier-based exploration [1] which is also the foundation of most of the current exploration algorithms. The basis of this method are frontiers, which represent the regions on the boundary between open space and unexplored space. This approach proposes that moving to the frontiers the robot will gain as much information as possible.

**Figure 1:**
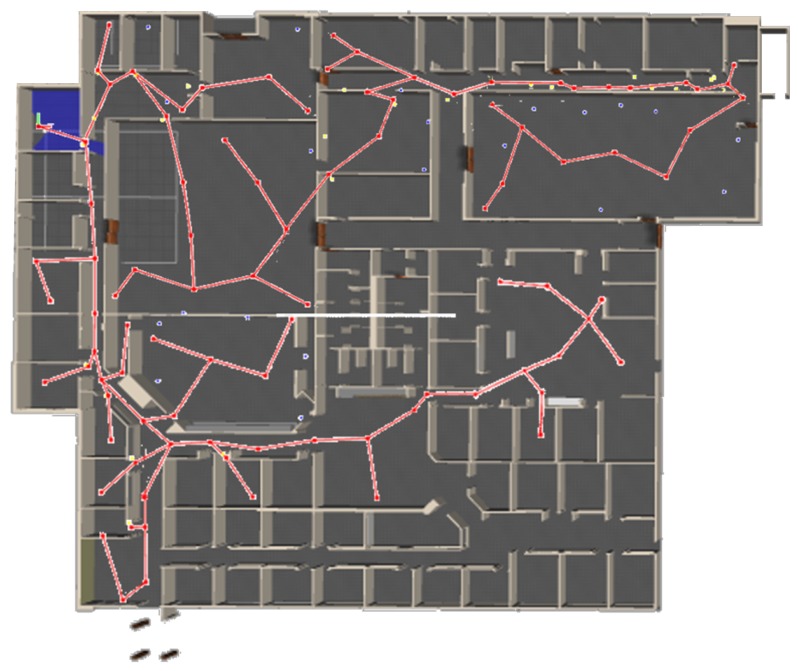
Result of autonomous exploration and mapping of the Willow Garage office environment using the proposed method. The resulting graph for the mapped area is overlapped over the Gazebo environment.

In this work, we propose a mobile robot exploration algorithm that combines frontier-based concepts with behavior-based strategies for indoor environments. A new frontier selection criterion is proposed based on the acquired geometric, topological and semantic information. Our aim is to autonomously build a topological representation of the environment to be used for further applications. An example of a map using the proposed method is shown in Figure 1, where the robot autonomously explored and mapped part of Willow Garage office environment. In this work, we focus on structured indoor environments but the method is applicable to more general environments. The main contributions of this work can be summarized as:

- Semantic classification of frontiers based on geometric characteristics
- A cost–utility function to drive the exploration strategy that takes into account geometric, topological and semantic information
- A probabilistic loop closure algorithm that determines when an area is reachable from two different paths

This paper is structured as follows. Section 2 surveys related work and concepts on exploration strategies. The proposed exploration algorithm is presented in Section 3; a detailed explanation of the different processes involved in the exploration is also presented. The complementary loop closing algorithm is explained in Section 4. Finally, the experiments validating the proposed work and the conclusions are presented in Section 5 and Section 6.

## 2. Related Work

Mobile robot exploration has attracted researchers attention since the beginnings of mobile robot developments as it is the way to autonomously build a representation of the environment. First studies on the topic based their representation on acquiring geometric precise information such as Configuration Space [2], Grid models [3] or Voronoi models [4]. Some researchers [5] noticed that the complexity of the problem increased exponentially as larger environments were considered when dealing with geometric maps and started to represent the information as topological maps. Others used the addition of external markers to help the exploration processes, such as Dudek et al. [6], who designed a robotic system that could identify, put down and pick up some markers that were used to guide the exploration.

Kuipers et al. [7] stated that previous works in exploration and map-building represented the environment as a geometrical map and some of them abstracted a topological graph afterwards to reduce computational costs in future stages. They proposed an exploration algorithm that directly obtained the topological representation. This system recognized qualitative properties as distinctive places and travel edges between them leading to a topological representation where geometry is assimilated into local descriptions of places and edges. Other authors also gave importance to topological representations; points of interest were identified as the robot moved in [8]. A virtual bubble was built around the robot occupying all the space perceived by the robot. The exploration finished when the bubble had occupied all the environment. Points of interest can be understood as a precursor of the frontier concept presented by Yamauchi [1]. Frontiers are regions on the boundary between open space and unexplored space. Yamauchi proposed that moving to the frontiers the robot will gain as much information as possible. In this first approach to the frontier concept, the selection of the best frontier to move corresponded to the nearest frontier. Frontier-based exploration has become one of the most used and robust exploration algorithms and many authors have based their exploration algorithms in the frontier concept. In [9], the authors added the information known about navigation and localization to the decision process for the next frontier to visit. Their algorithm maximizes the global utility which consists of information utility (maximize the amount of new information that can be acquired), navigation utility (minimize the displacement to the place) and localization utility (minimize the localization error). Other authors [10] defined an exploration strategy through multi-objective optimization of separated features where the selected candidate frontier is the one that is nearest to the individual ideal values. In the same direction, in [11], multiple features are proposed to optimize the decision making process but the selected candidate corresponds to the one that maximizes a global utility function. Amigoni et al. [12] used frontier-based exploration to build geometric maps. In [13,14], comparisons of different exploration strategies are presented. In both works, the authors concluded that, depending on the application, the decision would vary but generally frontier-based exploration using only cost (moving to the nearest frontier) requires less time. On the contrary, using cost and utility, such as the work in [15], extensive knowledge of the environment is acquired more quickly, which is important in rescue and surveillance tasks.

Meanwhile, other authors worked on improving behavior-based approaches for exploration. In [16], a topological map called navigation chart containing the actions to move between distinctive places is presented. In [17], the authors presented a behavior-based control approach to build a topological map that establishes connections between rooms. A topological exploration strategy is also presented in [18], in which the behaviors are grouped in node detection, node matching and edge travelling using a Voronoi diagram.

Recently, other exploration strategies have been proposed, such as the work presented by Fermin-Leon et al. [19,20], where rooms are identified by topological segmentation of contours. Each room or region is associated with a node and they used Tarry maze-searching [21] algorithm to move through the environment. In [22], the authors proposed an exploration and rescue method based on Partially Observable Markov Decision Processes which directly incorporates uncertainty in the decision process. In [23], a multirobot exploration algorithm in which each robot auctions for the next positions to reach is presented. Based on the observed part of the environment, the system estimates the outer border of the environment by the convex hull of the observed map and infers the structure of the unknown area. In [24], random next best views are connected through a RRT algorithm. The selection of the next best view is performed with regards to the amount of visible unmapped area and with a penalization for high costs. This work deals with 3D mapping and 2D surface inspection and shows a better performance of RRT exploration compared to frontier-based exploration for fine-grained, complex and detailed mapping. However, in house or office environments, frontier-based exploration obtained a faster result.

Many recent works have presented variations of the frontier-based exploration algorithm. In [25,26], the authors developed a frontier exploration algorithm where the function to chose the best candidate depended on the localizability and uncertainty based on entropy. This algorithm leads to a conservative exploration strategy that maintains a good uncertainty value through loop closure and revisiting poses. Some works [27,28] have focused on frontier-based exploration to perform navigation in an unknown environment. In these works, robots do not build a representation of the environment or completely explore the environment, rather they collaboratively reach their desired positions within the environment. In [29], a method for online mapping through Gaussian Processes occupancy maps (GPOMs) is proposed. An algorithm to extract probabilistic frontiers from GPOMs is used as frontier detection. Frontier selection is performed based on information gain and path length, but just considering geometric information. They showed higher performance than standard frontier-based exploration for big indoor environments. Lately, frontier exploration has also been applied to multirobot exploration establishing a routing priority for the frontiers and the robots [30]. Other works with multirobot frontier exploration have also added semantic and scene information to the decision process in order to separate the trajectories of the robots [31] or to obtain a higher reward of certain kind of areas [32]. However, these methods only used semantic information for the exploration process and built grid maps where these information is not included.

In this work, an exploration strategy is presented that differentiates from the previous works in that it builds a lightweight and efficient map representation that contains geometric, topological and semantic information. This same information is taken into account to lead the exploration strategy and determine possible loop closures. Semantic information is considered with regards to the traversing of transit area and it is included in the decision process through the cost–utility function. The exploration algorithm combines frontier-based concepts with behavior-based strategies for indoor environments. The purpose of this work is to improve the efficiency with regards to distance travelled and execution time, to increase the robustness of exploration algorithms dealing with large environments and to build a lightweight topological representation of the environment that includes all the geometric, topological and semantic information for further navigation.

## 3. Exploration Algorithm

**Figure 2:**
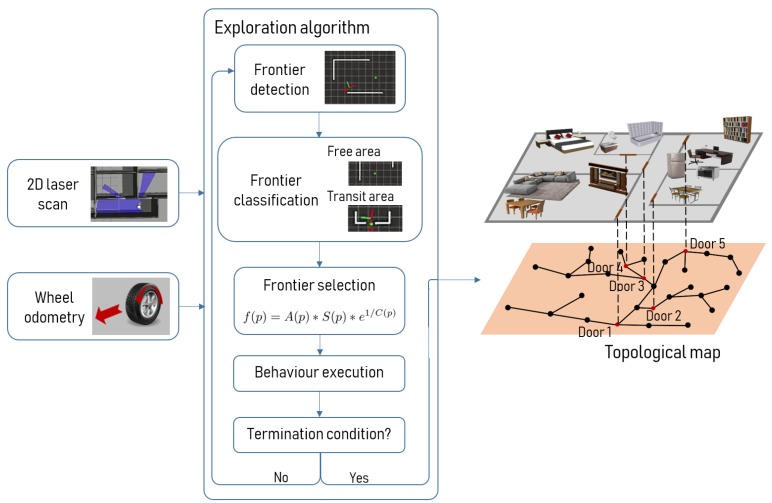
Processes involved in the exploration algorithm. The inputs of the system are the scans from a 2D laser and the odometry of the robot. The output of the system is the topological map of the environment.

The exploration algorithm proposed in this work is based on the frontier concept presented originally by Yamauchi [1]. According to that work, a frontier is a region on the boundary between free space and unknown space and moving to the frontiers the robot gains the maximum information for each movement. In our work, the first step for the robot is to detect the frontiers and decide where it is going to move to. A frontier is semantically classified as free area or as transit area depending on geometric information. Transit areas are defined as the frontier where the robot changes between two places (places can be rooms, roads, floors, etc.) and regardless of the size of the transit area the information gain that they offer is significant. This semantic information, along with the cost of moving to each frontier and the estimated utility, is used to determine the next best frontier. The robot is now ready to move to the desired position. To do so, it executes the behaviors corresponding to the current situation, as explained in Section 3.4. This process is executed iteratively until the termination condition is reached. The termination condition in our case is defined as the absence of interesting frontiers to visit, as explained in Section 3.5. Once the exploration algorithm has finished, the topological map according to the free space of the environment is completely built and can be used for further navigation. This process is illustrated in the diagram presented in Figure 2. Sensor information from a laser, a camera and wheel odometry are used as inputs to the system. When the exploration process has reached the termination condition, the topological map of the whole environment is available for further operations.

**Figure 3:**
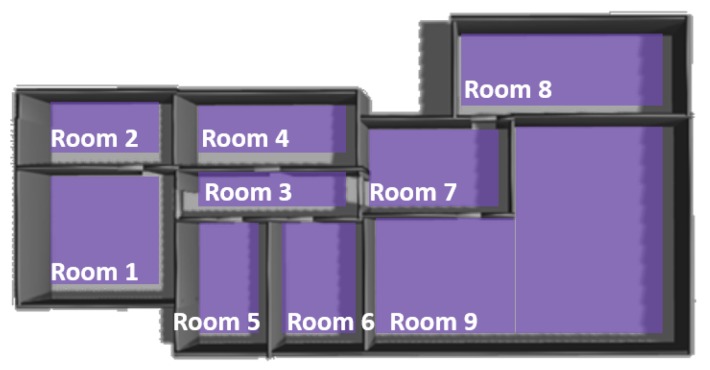
Simulation of house-like environment in Gazebo. The environment consists of nine rooms connected through 8 doors.

The purpose of this work is to give the capabilities to a robot to autonomously explore and build a representation of indoor environments, such as the one shown in Figure 3. This representation includes all the information that the robot has acquired from the environment. One of our aims is to use this algorithm in the future to help elderly people as a robot can learn the structure of their house and help them in daily-life activities. In the following subsections, each step for successfully performing the exploration algorithm is explained: frontier detection, frontier classification, frontier selection, the behavior-based strategy to reach the selected frontier and the termination condition that determines when the environment is fully explored.

### 3.1. Frontier Detection

Frontier detection is the process of detecting the boundaries between free-known space and unknown space. To perform this detection, the robot is equipped with a 2D laser sensor (Hokuyo URG-04LX). This sensor covers the surrounding area with a maximum radius of 5 m approximately. We consider that every group of laser measurements is a frontier if one of the following:

- The value of all the measurements corresponds to the maximum range value. This means that in that direction there is no obstacle in the seen region.
- It represents a significant gap between measurements. Even though range values do not reach the maximum value, it is possible to have occlusions between obstacles which are recognized through a significant difference between consecutive scans.

**Figure 4:**
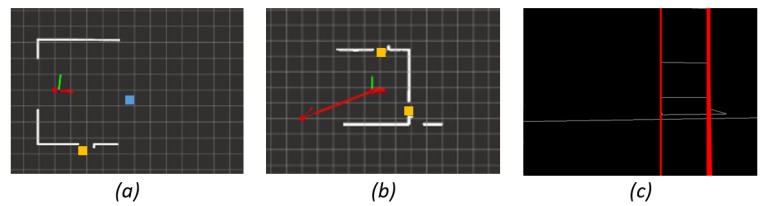
Semantic frontier classification: (**a**) a free area and a transit area are detected; (**b**) two transit areas, one of them is successfully detected as door despite the detection of a wall behind it; and (**c**) door frames detection based on Hough Transform is shown.

Once the frontiers are detected, we characterize them using the middle point (position to be reached afterwards) and the size of the frontier (that corresponds to the geometric utility). In Figure 4a,b, different frontiers have been detected and the position to be reached within the frontier is marked with a square.

### 3.2. Semantic Frontier Classification

Frontiers are semantically classified as free area or as transit area based on their geometric characteristics. In this work, transit areas are identified with doors (but they could be defined differently for other approaches or environments). Free areas relate to frontiers within a room and transit areas relate to frontiers that connect to different rooms (doors).

A preliminary door detector has been developed in this work, in which only the geometric characteristics obtained from the laser and a depth camera are taken into account. For a frontier to be considered a door, its size must correspond to a typical doorway between two coinciding segments. In Figure 4a,b, the robot identified three frontiers characterized as transit area or door (yellow) and one as free area (blue). In addition, in Figure 4b, an example of a frontier where there is a gap between measurements is shown. In this case, although the wall across the door is detected, the frontier is considered as the doorway. Once the laser information selects a gap as a possible door, camera information is used to confirm that hypothesis. A simple vertical line detector using Hough Transform was implemented. Vertical lines must be found close to the door frame in order to finally consider that gap as a door. In Figure 4c, the detection of the door frames of a door is shown.

Behaviors to check and confirm that a frontier detected as a transit area is really a transit area are executed in order to solve the misclassification that could occur in corners or dead-ends. As explained in Section 3.4, these behaviors are approaching to the center of the door and stopping 90 cm before reaching it. From that new position, the door detection algorithm is run again and it will confirm or discard that the frontier is a transit area.

Frontier classification was executed from 50 different random positions leading to the results shown in Table 1.

The results obtained show the good performance of the classifier in the detection of doors considering the high percentages of accuracy and sensitivity. In 92.98% of the cases, the classifier detects the doors correctly. In addition, the misclassification rate is quite low, only 7.02%. Failing situations were mainly due to dead ends or corners and sharp angles to doors.

This semantic classification between free and transit areas plays an important role in the exploration algorithm as it is one of the key points for the decision process.

### 3.3. Frontier Selection through Cost–Utility Function

A cost–utility function is implemented to decide the next best frontier to be visited. A cost–utility function is defined as the function that the system tries to maximize as it represents the most optimal value. In this case, the cost–utility function, *f(p)* for a given frontier *p*, is shown in Equation (1) and results from the combination of:

- The geometric utility, *A(p)*, corresponds to the size of the frontier. Bigger frontiers will offer a bigger range to acquire new information of the environment.
- The semantic utility, *S(p)*, gives a semantic importance to the transit areas. Despite its small size, transit areas open to a new space that will make the robot gain valuable new information.
- The topological cost, *C(p)*, refers to the topological distance that the robot will have to travel to reach the frontier. This cost is associated to the connectivity between frontiers. Consecutive frontiers will have a cost value of 1. However, if to reach one frontier the robot has to pass by other frontiers, its cost value will correspond to n+1, where *n* is the number of frontiers to cross.
  (1) $$f\left(p\right)=A\left(p\right)*S\left(p\right)*e^{1/C(p)}$$

In Equation (1), different utilities are related linearly, whereas between utility and cost the relation is a reverse exponential. Using this relation, we are penalizing the transitions that are not directly connected to the current frontier, as they imply path planning and several transitions. As observed in Table 2, there is a great penalization between cost value 1 (0%) and cost value 2 (39.35%) to avoid excessive path-planning and revisiting of nodes. Among higher costs, lower penalization increments are applied as the cost increases (as once the robot is performing path planning, the number of revisited nodes is not so determining). This effect is due to the reverse exponential related to the cost.

The main advantage of the cost–utility function proposed in this work is that it balances between distance and information gain. Information gain is considered with regards to frontier size and type. This can lead to situations where, although having small adjacent frontiers to visit, the robot plans a path to a more distant frontier that offers greater information gain.

The cost–utility function is calculated at each exploration iteration for each possible unvisited frontier and the best frontier corresponds to the one that maximizes the cost–utility function.

**Figure 5:**
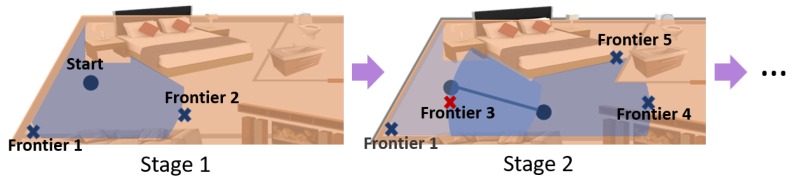
Sequence for the detection, classification and selection of frontiers in two exploration stages. Frontiers are analyzed and visited until the termination condition is met and the environment is fully explored.

Section 3.1, Section 3.2 and Section 3.3 can be summarized as the sequence presented in Figure 5. From a starting position, the robot seeks for the best option to start exploring the environment. Two frontiers have been detected and semantically classified as free area. Both frontiers are placed at a cost value of 1 and the geometric utility of Frontier 2 is much bigger than the one of Frontier 1. For this reason, in the first stage, the robot decides to move to Frontier 2. When the required position for that frontier is reached the second exploration stage starts. The robot detects three new frontiers. Frontier 3 is discarded as it corresponds to an already-visited area and Frontiers 4 and 5 are evaluated along with Frontier 1 that was not explored in the previous stage. Frontier 1 has a cost value of 2 and the new frontiers a cost value of 1. In addition, Frontier 4 opens to a much wider area, thus Frontier 4 will be the next one selected to explore. This sequence will continue until the termination condition is met.

### 3.4. Behavior-Based Exploration Strategies

The robot can perform three different behaviors to fulfill the exploration process. The behaviors implemented are *Move to next best view*, *Approach transit area* and *Cross transit area*. *Move to next best view* behavior is performed for frontiers classified as free area and it reaches the middle point of the next best frontier. *Approach transit area* and *Cross transit area* are performed for frontiers classified as transit area. The robot first performs *Approach transit area*, which consists on moving towards the middle point of the frontier but stopping 90 cm before reaching it. When that approaching position has been reached, the robot checks that it is effectively a transit area, executing again the door detection algorithm. If the transit area has been checked, the behavior *Cross transit area* is executed. It moves the robot through the transit area and beyond until it has entered the new room. If the transit area has been discarded, a new frontier to visit is sought. Each behavior requires different speed and precision conditions.

When the next best frontier is situated in a topological cost higher to 1, prior to executing the required behaviors the robot has to plan the path to reach the next best frontier. This path planning is performed using Dijkstra path planning algorithm that finds the shortest path between the known nodes of the environment and executes the corresponding behaviors for each of the nodes it has to traverse.

### 3.5. Termination Condition

The termination condition of the algorithm is the absence of any interesting frontier to visit. A frontier is considered interesting if its cost–utility value is higher than an experimentally defined value. If none of the remaining possible frontiers has a cost–utility value higher that the minimum considered interesting. the algorithm finishes. This procedure avoids long time explorations that lead the robot to areas that do not add extra information of the environment (such as corners or small dead-ends).

**Figure 6:**
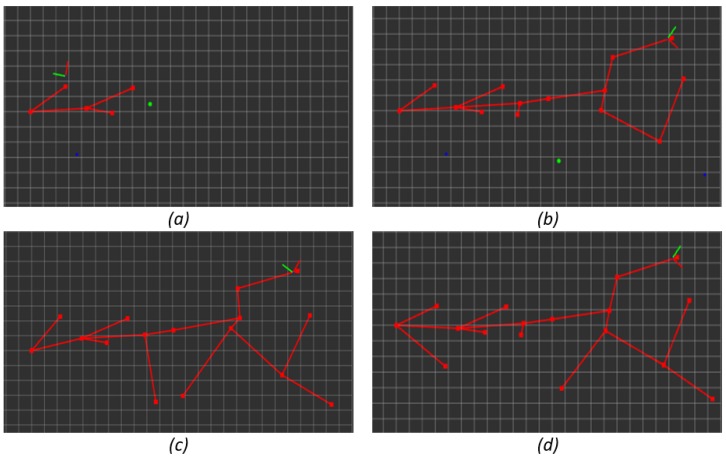
Topological map for different minimum interesting function values: (**a**) minimum value 80; (**b**) minimum value 40; (**c**) minimum value 30; and (**d**) minimum value 10.

The minimum interesting function value has been determined experimentally in the simulated indoor environment shown in Figure 3 in order to determine the highest value that allows full exploration of the environments without over-exploring it. The chosen value for the minimum interesting function is the same for the all experiments shown in this paper. In Figure 6, the resulting topological map for different minimum interesting function values is shown. Figure 6a was performed with a minimum value of 80; Figure 6b with a minimum value of 40; Figure 6c with a minimum value of 30; and Figure 6d with a minimum value of 10.

**Figure 7:**
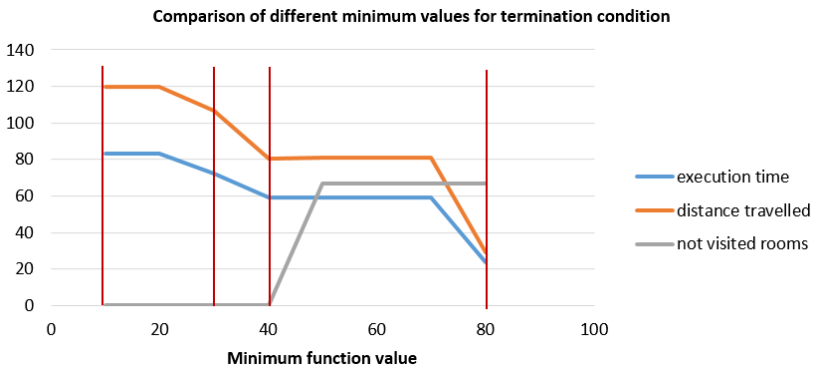
Comparison of the topological maps obtained for the different minimum interesting values (*x*-axis for the function value and *y*-axis for the metrics): traveled distance (m), execution time (min) and percentage of non-visited rooms.

Some of the differences can be observed at first sight, for example it is obvious that the exploration for Figure 6a, minimum value 80, is not complete, but some other differences are not so obvious without some metrics. The metrics used for determining the minimum interesting function value are the execution time, the distance travelled and the percentage of non-visited rooms. As seen in Figure 3, this environment consists of nine rooms and it is essential that the exploration algorithm explores each of the nine rooms. In Figure 7, the execution time (min) is shown in blue, the distance travelled (m) is shown in orange and the percentage of non-visited rooms is shown in grey. The minimum value must guarantee that all the rooms of the environment are visited, whihc corresponds to a minimum value equal or inferior to 40. Analyzing the execution time and the distance travelled, both are minimized in 40 (within the valid values according to the number of visited rooms), thus 40 will be the optimal value. However, we decided to set the value to 30 penalizing the distance travelled but setting a tolerance for other situations. From now on, all experiments took place with a minimum interesting function value for termination of the exploration of 30.

### 3.6. Topological Map Building

Through this exploration algorithm, a topological graph of the free space is built. Each visited frontier corresponds to a topological node and the behaviors for travelling between frontiers correspond to the topological edges. The semantic information for classifying the frontiers is stored in the node along with its geometric information and topological information (connectivity between nodes). Previous works have presented different topologies and node/edge association to different elements in the environment according to the task. The proposed method has the advantage of representing the traversability of the environment, unlike the work of Fermin-Leon et al. [19], where just a higher-level graph representing the connection between rooms is considered. In our work, traversability is represented as straight path lines between nodes where the distance between the nodes is sensor related so, when path planning, the robot could know if the path to reach the next node is free or occupied. Other topological representations, such as Generalized Voronoi Diagrams (GVD) [4], also refer to the traversability of the environment. However, these representations may include curved paths between adjacent nodes (more difficult trajectory following) and distances higher than the sensor range to reach the next node (the robot is more prone to take unsuccessful paths). In addition, topological maps based on objects or distinctive places (see, e.g., [7]) represent the connectivity or travel path between the mapped objects or distinctive places. As for GVD, distances between distinctive places might be larger than the sensory horizon of the robot. To summarize, the main advantages for navigation of the proposed topological mapping method is that it represents the traversability of the environment thought straight lines with a sensor-related distance to improve node-reaching capability.

**Figure 8:**
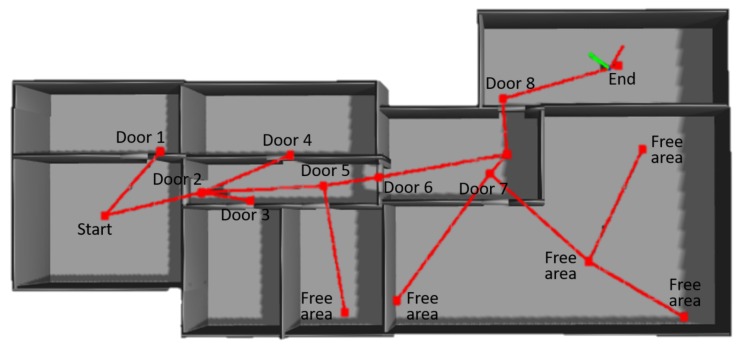
Example of a topological map build with exploration in a simulated environment. Red lines correspond to edges and red dots to nodes.

In Figure 8, the topological map of a given environment built through the proposed method is shown. As described above, the map represents the traversability and allows the robot to travel through all the environment. In further stages this representation will be used to plan trajectories and move autonomously according to the extracted information.

The exploration algorithm proposed up to now explores and builds a topological representation of indoor non-cyclic environments identifying the free areas and the transit areas. In the next section the loop closing strategy to successfully explore and build cyclic maps is explained.

## 4. Loop Closing Strategy

**Figure 9:**
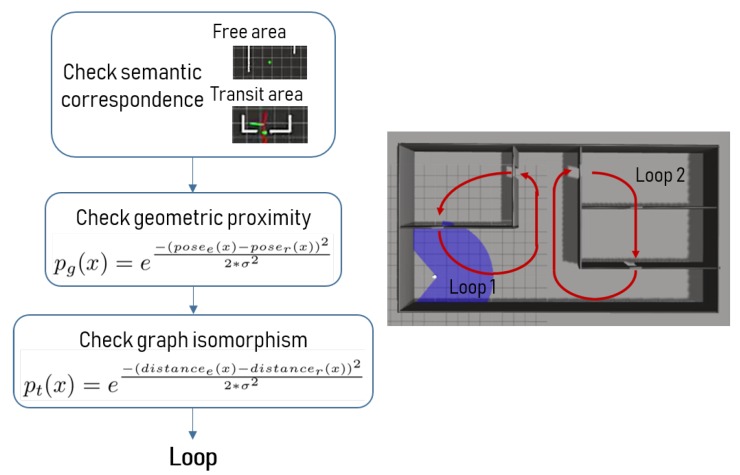
Simulated environment with two loop situations: one connecting two rooms and the other one connecting three rooms.

Robots will have to face any loop or cyclic situation (kitchens with two entrances or other office environments) at some point, as in the situation shown in Figure 9. This exploration algorithm is complemented with a Loop closure strategy to successfully build the maps and explore the environment even when cyclic situations appear. Loop closure has been widely addressed by researchers. Most of SLAM approaches only considered geometric conditions to perform loop closure [20] although some authors added visual features to the geometric information in order to have more robust results [33,34]. Other authors have tackled loop closure through localization. In [35], a probability distribution is maintained during the exploration and whenever there are two peaks in the distribution (two nodes with high probabilities) the algorithm tracks those nodes to look for a convergence, loop, or divergence, different locations. Another approach [36] is maintaining a tree with every possible hypothesis. Each hypothesis is associated with its probability and the tree is pruned until a decision is taken.

In this paper, we propose to use the geometric, topological and semantic information available for the nodes to estimate the uncertainty of being in a loop. We consider that the robot is in a loop when it is visiting (or closely visiting) a node that it has already mapped. According to the proposed algorithm this situation only happens when a node is reachable from two different paths from the same starting position. The process for identifying and accepting a loop is described in Figure 9.

In this work, loop probability is built from geometric, semantic and topological information. Firstly, geometric uncertainty is considered as the difference between the estimated position of a node and the current position of the possible loop node. Semantic information is considered as the condition for the original node and the loop node of belonging to the same semantic type (free area or transit area). Geometric loop probability, $p_{g}\left(x\right)$ has been computed using a Gaussian distribution, according to Equation (2). Terms $pose_{e}$ and $pose_{r}$ refer to the estimated and real positions, respectively, and σ refers to a variance value that has been experimentally tuned.

(2) $$p_{g}\left(x\right)=e^{\frac{-{\left(pose_{e}\left(x\right)-pose_{r}\left(x\right)\right)}^{2}}{2*\sigma^{2}}}$$

In the case that two semantically identical positions obtain a geometric loop probability greater than a threshold (experimentally set to 0.3), a graph isomorphism process starts in order to obtain the topological uncertainty. The graph isomorphism used in this work consists in replicating the morphology of the graph associated to the original node starting from the possible loop node. In this process, the first step is to verify that the prior of the original node is reachable from the loop node. A prior is considered reachable when there is no obstacle between the current position and the prior position. If it is reachable, the robot moves until it reaches the prior node. The estimated distance and the real travelled distance are compared to determine the topological loop probability $p_{t}\left(x\right)$ (Equation (3)). Terms $distance_{e}$ and $distance_{r}$ refer to the estimated and real distances to the prior node, respectively, and σ refers to a variance value that has been experimentally tuned.

(3) $$p_{t}\left(x\right)=e^{\frac{-{\left(distance_{e}\left(x\right)-distance_{r}\left(x\right)\right)}^{2}}{2*\sigma^{2}}}$$

Graph isomorphism process iterates and compares the graphs until a positive or negative decision is reached according to the global loop probability, $p_{loop}\left(x\right)$. Normalized global loop probability, Equation (4), relates geometric and topological uncertainties.

(4) $$p_{loop}\left(x\right)=p_{g}\left(x\right)*\pi \left(p_{t}{\left(x\right)}_{i}\right)$$

A positive decision implies the acceptance of the loop and the update of the topological map to this new situation. A positive decision is reached when the global probability surpasses a threshold value (set to 0.9) within five iterations of graph comparison. A negative decision implies that the loop is rejected and the topological map is not modified. A negative decision is reached when the global probability does not surpass the threshold value within five iterations, or when any of the priors is not reachable from the looping or isomorphic nodes.

In the following section, simulation and real-world experiments for non-cyclic and cyclic environments are included to uphold the improvement due to the proposed method.

## 5. Experimental Results

### 5.1. Experimental Setup

Exploration experiments were conducted firstly using Gazebo simulation environment. The performance and resulting maps were visualized using RViz as the work was developed using ROS (Robot Operating System framework) and C++. Several indoor environments and objects were created to develop the experiments as close as possible to real situations. The simulated version of Turtlebot 2 robot is equipped with the simulated sensors Hokuyo URG-04LX laser sensor and Asus Xtion Pro camera to perceive the environment. Regarding the computational resources, for both simulated and real-world experiments, a PC with IntelCore i7-6500U CPU@2.50Hz 12GB RAM was used.

Real-world experiments in a house environment are also presented. Turtlebot 2 robot was used for the experiments and RViz visualizer was used to observe the resulting map of the environment. As in the simulated experiments, Turtlebot 2 is equipped with a Hokuyo URG-04LX laser sensor and an Asus Xtion Pro camera. The robot’s speed was limited to 0.4 m/s in linear velocity and 0.7 rad/s in angular velocity.

For all the experiments presented in this paper, the same set of parameters was used (maintaining the same configuration also for the compared algorithms). The employed parameters are summarized in Table 3.

### 5.2. Results for Medium-Size House-Like Environments

Exploration was tested in two different non-cyclic simulated house-like environments, one medium-size house environment (130.5 m${}^{2}$) and one big-size house environment (235 m${}^{2}$). In the case of the medium-size house environment, experiments were performed with and without furniture to compare the resulting topological maps.

**Figure 10:**
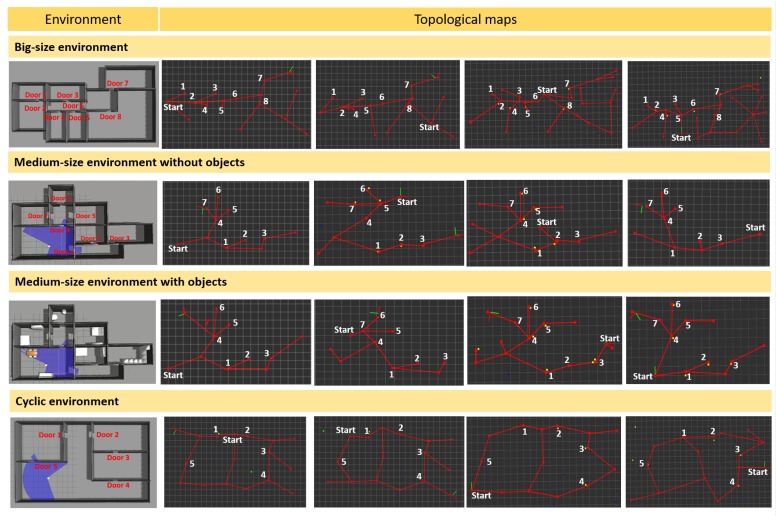
Result for simulated non-cyclic and cyclic indoor environment. The first row shows the big-size non-cyclic simulated environment and the topological maps obtained from different starting positions. The second row shows the medium-size non-cyclic simulated environment without furniture and the topological maps obtained from different starting positions. The third row shows the medium-size non-cyclic simulated environment with furniture and the topological maps obtained from different starting positions. The fourth row shows the cyclic indoor simulated environment and the topological maps obtained. Both loops were recognized for every initial position as represented in the maps. Correspondences between doors and starting positions are included to facilitate the interpretation of the structure.

The big-size house environment and the resulting topological graph from the exploration are shown in Figure 10 (first row). Four resulting topological graphs from initial random positions are shown. Initial positions and the nodes corresponding to doors are included with the graph for clarity reasons. A similar structure is observable in the graphs and the tested environment. Differences are due to the difference in the initial position. In both cases, the eight doors were detected and all the rooms were explored.

Regarding the experiments for the medium-size house environment, results are shown for the environment without furniture (Figure 10, second row) and with furniture (Figure 10, third row). Four resulting topological graphs from initial random positions are shown. Initial positions and the nodes corresponding to doors are included with the graph for clarity reasons. In this case, independent of the presence or the absence of furniture, the generated maps are very similar and representative for the environment. All rooms were explored in the four cases and the influence of objects in the exploration process and resulting maps is minimal. The first graph for the environment without and with furniture was performed from the same initial position to exemplify the similarity between the graphs.

In the three cases shown in the first three rows of Figure 10, environments without cyclic situations were explored and mapped. The resulting topological maps represent their corresponding environment and they allow full exploration of the environment. Although different starting positions result in different graph structures, the topological maps obtained faithfully represent the environment.

### 5.3. Results for Cyclic Indoor Environment

Experiments in a simulated cyclic indoor environment were carried out to check the proposed loop closing algorithm. The environment (342 m${}^{2}$) has two loops, one connecting two rooms together and the other one connecting three rooms, as shown in Figure 10 (fourth row). Topological maps were created from different starting positions; all of them successfully mapped the environment and closed the two loops. Examples of topological maps are shown in Figure 10 (fourth row). The two loops are observable in the resulting graphs and the robot explored the whole environment and detected all the doors. Average execution time for the experiments was 52.5 min and average traveled distance was 99.43 m. The loop connecting Door 1 and Door 5 was successfully closed after two iterations of graph isomorphism and the loop connecting Doors 2–4 was successfully closed after 3.75 iterations (average values).

### 5.4. Results for Big Office Environments

Exploration was also tested in two more complex and bigger simulated office environments. These environments are composed of more rooms of very different sizes and include several loops.

**Figure 11:**
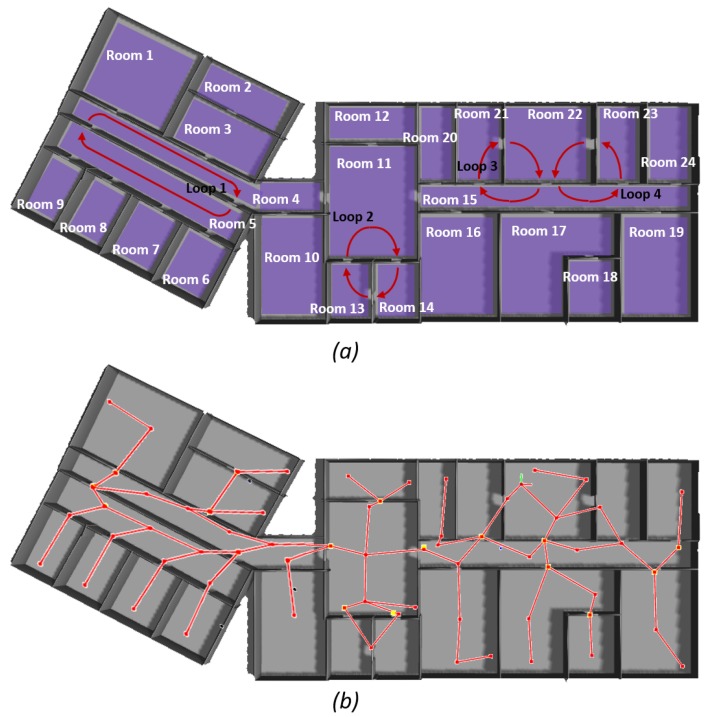
Results for the first big office simulated environment: (**a**) the environment and the different rooms and loops are indicated; (**b**) the resulting topological map. The topological map is layered on top of the simulated environment in order to facilitate reader’s comprehension.

The first office environment (1141.26 m${}^{2}$) is shown in Figure 11a. It is composed of 24 rooms and it includes four simple loops. In Figure 11b, the resulting topological map after exploration is shown. The robot explored the whole environment, visited all the rooms and closed the four loops. The topological map consists of 67 nodes and 69 edges. Twenty-seven nodes were recognized as transit areas and 40 as free areas.

**Figure 12:**
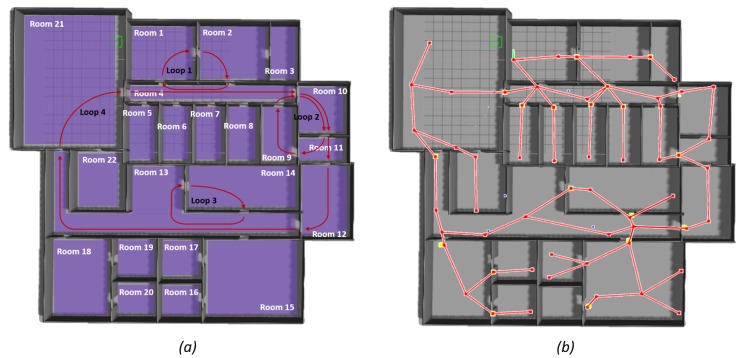
Results for the second big office simulated environment: (**a**) the environment and the different rooms and loops are indicated; and (**b**) the resulting topological map. The topological map is layered on top of the simulated environment in order to facilitate reader’s comprehension.

The second office environment (1137.4 m${}^{2}$) is shown in Figure 12a. It is composed of 22 rooms and it includes four loops, one of them connecting a large area of the environment (Loop 4). In Figure 12b, the resulting topological map after exploration is shown. As observable, the robot explored all the rooms in the environment and closed the four loops successfully. The topological map consists of 61 nodes and 64 edges. Twenty-five nodes were recognized as transit areas and 36 as free areas.

### 5.5. Comparison with Other Exploration Strategies

To validate the results obtained for non-cyclic indoor environments, a comparison to other state-of-the-art algorithms is presented. The proposed algorithm was compared to: wall-following exploration [37], frontier-based exploration using cost function [1] and frontier-based exploration using GB-L cost–utility function [15]. Wall-following algorithm consists in simply following the walls until the starting point is reached again. This algorithm is not valid for cyclic environments as it would explore just one of the loops. Regarding frontier-based exploration using a cost function, we used the same structure as for the proposed algorithm but the cost function is adapted to meet the new requirements. The cost function for this algorithm is shown in Equation (5), where L(p) refers to the topological length to reach the frontier.

(5) f(p)=λ/L(p))

Finally, frontier-based exploration using GB-L cost–utility function works with the cost–utility function included in Equation (6). It is a well-known and robust definition for frontier-based exploration where A(p) refers to and estimate of the unexplored area visible from p, λ weighs the new information and the cost and L(p) refers to length of the path (as only topological distance is considered in this work, L(p) refers to connectivity and not geometrical length).

(6) f(p)=A(p)∗exp(−λ∗L(p))

The four exploration strategies were implemented and their performances were compared according to average execution time and average distance traveled for the middle-size environment without objects shown in Figure 10 (second row). Execution time and Traveled distance were computed for 10 exploration sessions for each algorithm from random initial positions. Average results are shown in Table 4. All the exploration strategies were asked to build a topological map of the environment as map representation and they were evaluated under the same conditions (removing non-interesting frontiers and same termination condition).

Frontier-based exploration algorithms had a much better performance than wall-following, which could be predictable. The experiment that led to a lower execution time was GB-L function frontier-exploration and the experiment that traveled a lower distance was the cost function frontier-exploration. However, the proposed algorithm gave the overall best performance considering both average execution time and average traveled distance.

### 5.6. Results for Real-World Indoor Environments

**Figure 13:**
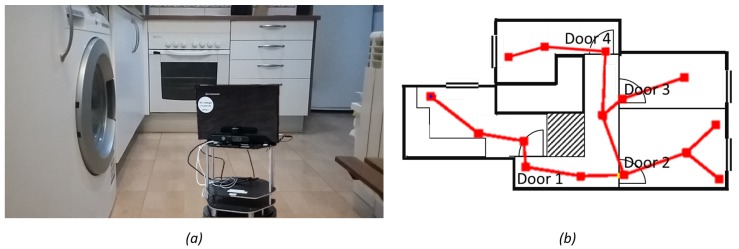
Real experiment in a house environment: (**a**) Turtlebot robot during the exploration process; and (**b**) the topological map of the real house-like environment generated with the exploration. The topological map consists of 15 nodes (4 of them classified as transit area and 11 classified as free area) and 14 edges.

Experiments in a house were developed using Turtlebot 2 robot to verify the viability of the proposed method in real life. Experiments were performed in an empty house of approximately 60 m${}^{2}$ consisting of five rooms (four rooms and a corridor). In Figure 13a, the robot is shown in the real house environment. The performance of the exploration algorithm and the map building is shown in the attached video (see Supplementary Materials) and the resulting map of the environment is shown in Figure 13b. A schematic view of the environment is shown along with the generated graph. The graph is built with 15 nodes (four of them classified as transit areas) and 14 edges connecting them. The environment was totally explored in 12 min and the robot travelled 34.5 m.

## 6. Conclusions and Future Works

In this work, we have developed an exploration algorithm based on frontier-based exploration and behavior-based strategies that builds a topological map of the environment. We proposed using semantic, geometric and topological information of the environment to determine the next best position to visit in indoor environments through a cost–utility function. We also proposed a probabilistic loop closure method that uses those sources of information to validate the loops and solve the correspondences in the map. The novelty of the proposed exploration method is the semantic frontier classification and the cost–utility function for frontier selection. This semantic frontier classification is not an accessory process as it takes part in all the steps to successfully perform the exploration (frontier selection, behavior selection and loop closure). Semantic frontier classification divides the environment into transit areas and free areas. In this work, doors were used as transit areas but other approaches could be considered. For example, in outdoor road environments, transit areas can be road intersections. In this way, if the robot (or autonomous car) is moving on a road, it will map all the nodes along the road path as free area until an intersection is reached where a transit area will be defined. This will isolate the road segments as different connected places. The proposed exploration algorithm could be adapted to other scenarios just including the corresponding transit area detector and some specific constrains (as exploring within the road track limits).

We propose a topological mapping of the environment due to its robustness and efficiency when dealing with large environments. We have extended the topological map with the geometric and semantic information that is relevant for the well-performance of the navigation.

Finally, we validated our complete system in simulated and real indoor environments. We showed that the proposed method performs better than other state-of-the-art methods in terms of execution time and traveled distance. We also presented different maps generated for different environments and their correspondence and fitness to them.

Future works include path-planning and navigation using the autonomously built map, allowing re-mapping for long-lasting consistency of the map and calculating automatically the thresholds and values for the exploration and loop closure processes. In addition, a study of the drift will be performed for exploration of large environments.

In conclusion, we demonstrated that including semantic, geometric and topological information in the exploration process and topologically mapping the environment improves autonomous exploration performance with regards to execution time, distance travelled and robustness for large environments and more efficient results are obtained in real time.

## Figures and Tables

**Table 1:** Results for Semantic frontier classification.

Measurement	Value	Measurement	Value
Prevalence	0.5263	Sensitivity	0.9333
Accuracy	0.9298	Specificity	0.9259
Misclassification rate	0.0702	F1-score	0.9333

**Table 2:** Study of the relation between cost and utility and the influence of cost in the proposed cost–utility function.

*C(p)*	*A(p)*	*S(p)*	*f(p)*	*% of Penalization*
1	60	1	**163.09**	0%
2	60	1	**98.92**	39.35%
3	60	1	**83.71**	48.68%
4	60	1	**77.04**	52.77%

**Table 3:** Set of parameters used for the experimental evaluation.

Parameter	Value	Parameter	Value
Maximum Laser Range	5 m	Semantic utility value for a transit area	80
Laser ranges corresponding to front	[275, 365]	Semantic utility value for a free area	1
Laser distance to consider front free	1.5 m	Geometric loop variance	0.9
Distance between successive scans to accept gap	0.8 m	Topological loop variance	0.85
Number of scans for minimum interesting gap	10	Loop probability to start loop closure routine	0.3
Small door size (single leaf)	[0.8, 1.2] m	Loop probability to accept loop	0.9
Big door size (two leaves)	[1.6, 2.4] m	Number of iterations to discard loop	5
Secure distance to approach and check door	0.9 m	Frontier reaching tolerance	0.1 m, 0.02 rad
Distance to cross door (after door)	0.5 m	Minimum function value for termination	30

**Table 4:** Comparison of different exploration strategies according to execution time and traveled distance: wall-following, frontier-exploration with cost function, frontier-exploration with GB-L function and the proposed method.

	Execution Time (min)	Traveled Distance (m)
wall-following	56	97.2
cost function	51	57
GB-L function	43	59.4
**proposed algorithm**	**44**	**57.5**

## Supplementary Materials

A video showing the results of one of the performed experimental tests is available online at https://youtu.be/ZKx37niBmt8.
